# Evaluation of the effects of *Uncaria rhynchophylla* alkaloid extract on LPS-induced preeclampsia symptoms and inflammation in a pregnant rat model

**DOI:** 10.1590/1414-431X20198273

**Published:** 2019-05-16

**Authors:** Liang-Zhi Wu, Xiao-Min Xiao

**Affiliations:** 1 Department of Obstetrics and Gynecology, 1st Affiliated Hospital of Jinan University, Guangzhou, Guangdong, China; 2 Department of Obstetrics and Gynecology, Guangdong Second Provincial General Hospital, Guangzhou, Guangdong, China

**Keywords:** Preeclampsia, Inflammation, Uncaria rhynchophylla, Alkaloids

## Abstract

Excessive pro-inflammatory cytokines result in adverse pregnancy outcomes, including preeclampsia-like phenotypes, and fetal growth restriction. Anti-inflammation might be an effective therapy. The aim of this research was to investigate whether *Uncaria rhynchophylla* alkaloid extract (URE), a highly safe anti-inflammation constituent of the herb, can inhibit inflammation and improve clinical characteristics of preeclampsia in a lipopolysaccharide (LPS)-induced preeclampsia rat model. The rat model was established by daily administration of LPS (1 μg/kg body weight per day) from gestational day (GD) 14 to 19. Different doses of URE (35, 70, and 140 mg/kg body weight per day) were administered from GD 14 to GD 19. The effects of URE on proteinuria, maternal hypertension, pregnancy outcomes, as well as pro-inflammatory cytokines levels in serum and placenta were measured. High-dose URE (HURE) treatment decreased LPS-induced mean 24-h proteinuria and systolic blood pressure, and increased fetal weight, placental weight, and the number of live pups (P<0.05). Moreover, increased serum and placental levels of interleukin (IL)-6, IL-1β, tumor necrosis factor-α, and interferon-γ in the LPS-treated group were obviously inhibited after HURE administration (P<0.01). URE improved preeclampsia symptoms and mitigated inflammatory responses in the LPS-induced preeclampsia rat model, which suggests that the anti-inflammation effect of URE might be an alternative therapy for preeclampsia.

## Introduction

Preeclampsia (PE) is a pregnancy-specific multisystem disorder and is characterized by proteinuria, hypertension, unexplained seizure, and severe headache (1). The manifestations of fetal PE include abnormal fetal growth, fetal oxygenation, and reduced amniotic fluid (2). This is a severe obstetric problem, of which the definite etiology is still unclear. PE affects about 5% of pregnancies and is a leading cause of maternal morbidity and mortality (3). Insulin resistance, increased systemic inflammatory response, and endothelial dysfunction are also present in PE (4). Although the main cause that triggers these dysfunctions in PE remains unknown, accumulating evidence shows that abnormal maternal-fetal inflammatory response and immune system activation play a significant role in the pathogenesis of PE (5). Alterations in maternal peripheral blood show that pregnancy is related to a mild yet sustained systemic inflammatory response (6). Recent research has also reported decreased serum levels of anti-inflammatory cytokines and increased serum levels of pro-inflammatory cytokines in PE women compared to normal pregnancy (7). Despite PE being considered an exaggerated inflammatory response resulting from a changed immune response, there is no effective and innovative treatment for PE.

Natural medicines have displayed positive effects on blood pressure and proteinuria in lipopolysaccharide (LPS)-induced PE rats (8). Similarly, administration of the extract from *Vitis labrusca* causes insulin resistance, a decrease in blood pressure, and increased the numbers of live fetuses in L-NAME-induced rats (9). Therefore, the search for herbal medicines with lower toxicity and efficient effects is important for developing novel therapy for PE treatment.

The herb *Uncaria rhynchophylla*, also known as Gou-teng, is an important traditional Chinese medicine generally used for the treatment of fever, bilious disorders, and dizziness for years in China. The leaves and stems of *U. rhynchophylla* are also used to treat nervous disorders and cerebrovascular diseases associated with epilepsy, hypertension, and preeclampsia (10). Indole alkaloids are the major active components of *U. rhynchophylla* extracts that have the anti-epilepsy, anti-hypertensive, and anti-inflammatory activities (11–13). *U. rhynchophylla* exhibits neuroprotective activities via the suppression of COX-2 and the subsequent decrease of inflammation in rats (14). Rhynchophylline, one of the main components of *U. rhynchophylla*, exhibits anti-convulsive and neuroprotection effects (15). In addition, *U. rhynchophylla* and rhynchophylline displayed anti-convulsive activities in kainic acid-induced rats via the suppression of interleukin (IL)-1β and brain-derived neurotrophin factor gene expressions (11). Moreover, *U. rhynchophylla* displayed inhibitory activities on LPS-induced IL-1β and NO production in macrophages (16). IL-1β and other inflammatory cytokines such as tumor necrosis factor-α (TNF-α), IL-6, and interferon-γ (IFN-γ) may be associated with the pathogenesis of PE (17,18).

LPS, a commonly known endotoxin, is a toxic component of Gram-negative bacterial cell walls, which has been linked to preeclampsia, fetal growth restriction, and embryonic resorption (19,20). Gram-negative bacterial infections are a cause of preterm labor and fetal loss in humans (21). Establishing maternal infection by injecting pregnant rodents with LPS led to an increase of inflammatory levels in the placenta and gave rise to preeclampsia, fetal loss, and fetal growth restriction (22). Hence, anti-inflammation might be an effective therapy for the prevention and treatment of these complications (23).

Therefore, the purpose of the present research was to investigate whether *U. rhynchophylla* alkaloid extract (URE) could improve PE-like symptoms in a LPS-induced rat model via suppression of pro-inflammatory cytokines. In addition, the alkaloids composition of URE was characterized using HPLC-MS methods. This study aimed to further characterize the possible functional components that are responsible for its anti-hypertensive and anti-inflammatory activities.

## Material and Methods

### Reagents and materials

Dried *U. rhynchophylla* was purchased from Guang Dong Feng Chun Pharmaceutical Co., Ltd. (China). Ultrapure LPS (strain *Escherichia coli* 055: B5), isorhynchophylline, yohimbine, 3α-dihydrocadambine, raubasine, hirsuteine, and hirsutine were purchased from Sigma-Aldrich (USA). Enzyme-linked immunosorbent assay (ELISA) kits specific for IFN-γ, IL-6, IL-1β, and TNF-α were purchased from eBioscience (USA).

### Preparation of URE

Dried *U. rhynchophylla* (500 g) was extracted four times with 500 mL of 50% ethanol. After filtration, the extract was evaporated and condensed under a vacuum condition. Next, the concentrate was dissolved in 50 mL water and added into the NKA-II macroreticular resin column for adsorption for 16 h. The column was washed with water and 20% ethanol, in order to get rid of impurities. URE was refined through elute with 95% ethanol. The eluate was collected and condensed in vacuum, and then further dried for 24 h at –20°C using a freeze-dryer (24).

### HPLC/MS analysis of URE

The alkaloids present in the URE were identified by high performance liquid chromatography-mass spectrometry (HPLC-MS) using an Agilent 1260 HPLC system equipped with an Agilent 6400 MSD (USA) with the electrospray source operated in the positive ion mode. Samples containing 0.5 mg/mL of URE in acetonitrile were passed through a 0.22 µm filter, and aliquots (1 µL) were injected onto Agilent Eclipse Plus C18 column (4.6×50 mm, 1.8 µm, 600 bar). Gradient elution was carried out using a mixture of 0.1% formic acid in water (A) and 100% acetonitrile (B) supplied at a flow rate of 0.4 mL/min. Detection was performed at 254 nm. The mobile phase was kept at 95% A between 0 and 0.5 min, followed by a linear gradient from 95 to 30% A between 0.5 and 5 min, and subsequently kept at 30% A between 5 and 10 min.

### Animal experimental design

The animal experimental protocols were approved by the Animal Ethics Committee of the Guangdong Second Provincial General Hospital and all animal studies were carried out according to Guide for the Care and Use of Experimental Animals. The 6–8-week-old female Sprague-Dawley rats (weighing 180–210 g) were purchased from the Experimental Animal Center of Hunan province and housed under laboratory temperature of 22–24°C, relative humidity 50–60%, illumination between 06:00 am and 06:00 pm with free access to basal diets and water. After a week of acclimatization, all female rats were mated with fertile males at a 1:1 ratio, and a positive vaginal smear for sperm defined gestational day (GD) 0. A total of 40 female rats were randomly divided into five groups (8 rats each group): pregnant control group (PC), LPS-treated control group (MC), LPS plus high-dose URE group (HURE, 140 mg/kg body weight per day), LPS plus medium-dose URE group (MURE, 70 mg/kg body weight per day), and LPS plus low-dose URE group (LURE, 35 mg/kg body weight per day). The experimental PE rats were induced by injection of LPS (1 μg/kg body weight per day, dissolved in 2 mL saline) on GD 14 (25). Intragastric administration was carried out once a day for all rats from GD 14 to 19. Rats in the PC group and MC group were intragastrically administered an equivalent volume of saline.

### Measurement of cytokines

On GD 20, blood samples were collected from the inferior vena cava for cytokine measurement. Afterwards, all rats were euthanized. The placentas and pups were weighed. All samples were kept at –20°C for further analyses. Total lysates were obtained by homogenizing 100 mg of placenta in 1000 μL of RIPA lysis buffer. The levels of serum and placental cytokines (IL-6, IL-1β, TNF-α, IFN-γ) were quantified by a commercial ELISA kit (Bio-Rad, USA) according to the manufacturer’s instructions. All samples were determined in duplicate. The data analysis was performed using Bio-Plex Manager (Version 5.0, Bio-Rad), and data are reported as concentrations (pg·mL^-1^·mg^-1^ or pg/mL).

### Measurement of systolic blood pressure (SBP) and proteinuria

On GDs 6, 10, 14, 16, and 18 (between 8 a.m. and 10 a.m.), the SBP was measured with a tail cuff sphygmomanometer (CODA, Kent Scientific, USA). All rats were warmed to 38°C for adapting to the experimental conditions before the actual measurements were performed. For each rat, the value of SBP was measured continuously 12 times, and three continuous values varying less than 6 mmHg were averaged to record the SBP. On GDs 7, 12, 17, and 19, each rat was housed in an individual metabolic cage to obtain the urine sample. Urine samples were centrifuged at 5000 *g* for 10 min at 22°C, and the supernatant was obtained for further analysis of the protein content. The protein level was measured with the pyrogallol red method (26).

### RNA isolation and real-time polymerase chain reaction (RT-PCR)

Total RNA was extracted from placenta tissues using TRIzol reagent (Invitrogen, USA), according to the manufacturer's instructions. cDNA synthesis was carried out by reverse transcription of 1 µg of purified RNA using First Strand cDNA Synthesis kit (Takara, China). RT-PCR amplification was performed with the SYBR Green qPCR Master Mix kit (Thermo, USA), following the manufacturer's protocol. The qPCR was performed in duplicate, using GAPDH as the reference gene. The condition of RT-PCR amplification reaction was as follows: 40 cycles of 95°C for 10 s, 60°C for 15 s, and 72°C for 30 s with the specific primer sequences (Table 1). Data analysis was carried out using the 2^-ΔΔCT^ method.

**Table 1.** Primer sequences for quantitative real-time RNA.

Genes	Forward primer	Reverse primer
IL-6	5′-TGGTGATAAATCCCGATGAAG-3′	5′-GGCACTGAAACTCCTGGTCT-3′
IL-1β	5′-GCCGATGGTCCCAATTACAT-3′	5′-ACAAGACCTGCCGGAAGCT-3′
TNF-α	5′-CCCTCACACTCAGATCATCTTCT-3′	5′-GCTACGACGTGGGCTACAG-3′
IFN-γ	5′-TGAGCCAGATTGTTTCGATG-3′	5′-TCCTTTTGAAACTCGGAGGA-3′
GAPDH	5′-CAACTTTGGCATTGTGGAAGG-3′	5′-ACACATTGGGGGTAGGAACAC-3′

### Immunohistochemistry staining analysis of the expression of NF-**κ**B p65

The collected placental tissues were fixed in 10% formalin and embedded in paraffin. The antigen retrieval of placental tissue sections (4-µm thickness) was carried out under high pressure after deparaffinization. Tissues were immersed in 0.3% hydrogen peroxide for 30 min to block the nonspecific antigens and endogenous peroxidase activity. Then, the tissue sections were incubated for 30 min with 5% goat serum, and subsequently incubated with rabbit anti-NF-κB p65 antibodies (1:50 dilution) in a humidified chamber overnight at 4°C. After washing with PBS, the sections were incubated with rabbit antigoat antibody for 20 min and were subsequently incubated with horseradish peroxidase and then counterstained with hematoxylin. The tissues were analyzed under a bright-field microscope. The degree of NF-κB expression was indicated as the percentage of nuclear NF-κB p65-positive cells out of the total number of placental cells.

### Statistical analysis

All experimental data are reported as means±SE. Data analysis among multiple groups was performed by one-way analysis of variance (ANOVA) followed by the least significant difference *post hoc* test or Dunnett's test. Data were analyzed by SPSS 19.0 software (SPSS, USA). P-values of <0.05 were regarded as statistically significant.

## Results

### Chemical characteristics of oxindole alkaloids from URE

**Figure 1.**
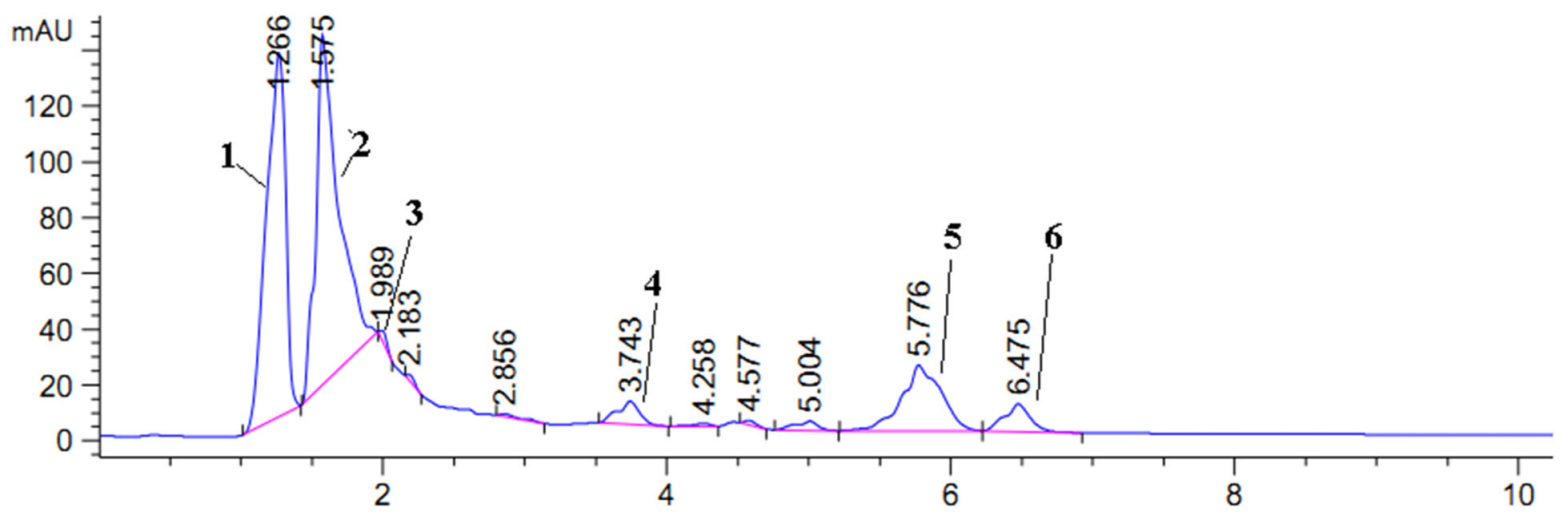
Chromatograms of alkaloids occurring in *Uncaria rhynchophylla* extract. 1: isorhynchophylline; 2: yohimbine; 3: 3α-dihydrocadambine; 4: raubasine; 5: hirsuteine; 6: hirsutine.

**Figure 2.**
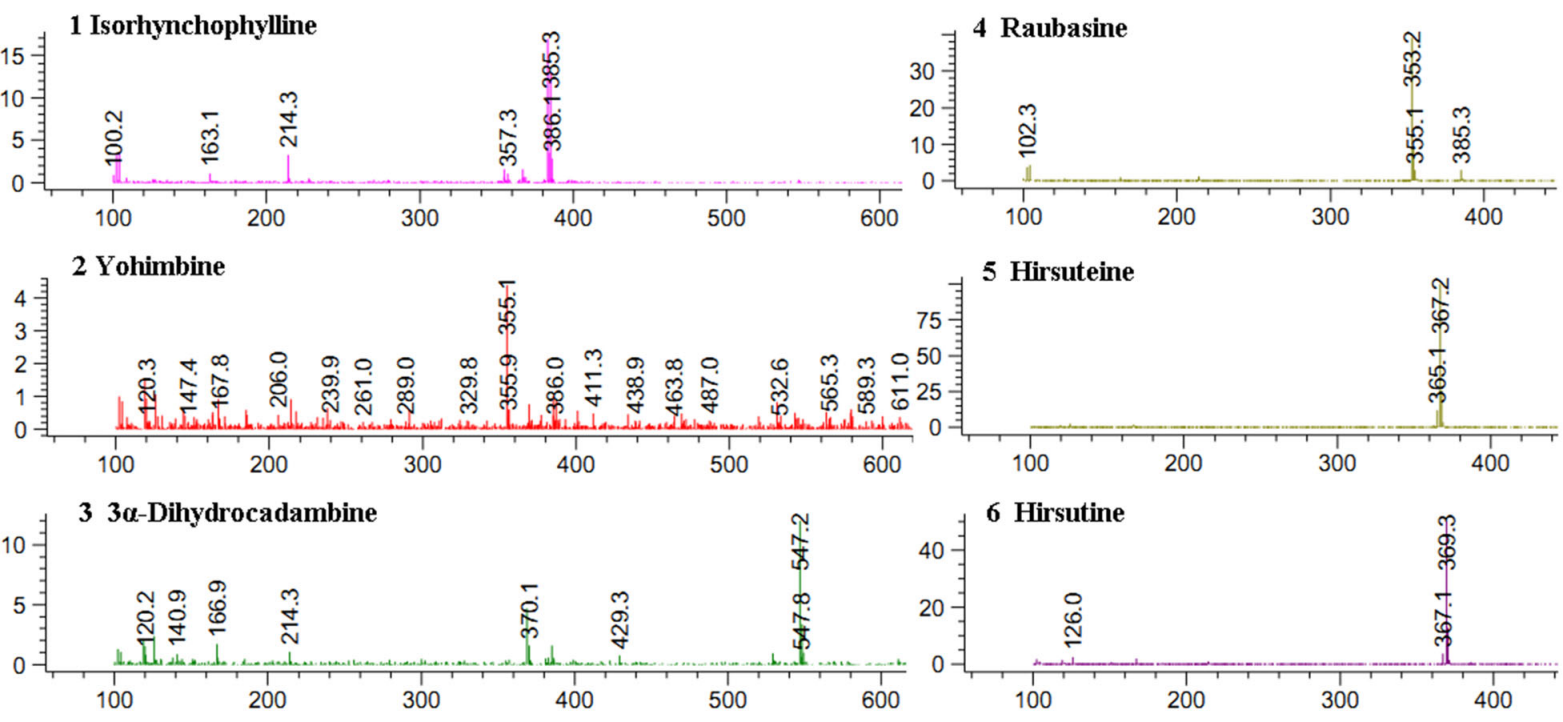
Positive fragments of alkaloids present in *Uncaria rhynchophylla* extract and characterization of six representative components assessed by HPLC-MS.

Firstly, the amounts of total oxindole alkaloids were quantified in order to standardize the URE. Of the URE total alkaloid content, 66.3% was of oxindole alkaloids. The chromatograms of alkaloids occurring in URE detected at 254 nm is displayed in Figure 1. The qualitative identification using standard reference solutions confirmed the presence of isorhynchophylline, yohimbine, 3α-dihydrocadambine, raubasine, hirsuteine, and hirsutine. The six constituents were supported by previous research (27). Detailed HPLC-MS data of these constituents are shown in Figure 2.

### URE reduced the hypertension in the LPS-induced PE-like rats

**Figure 3.**
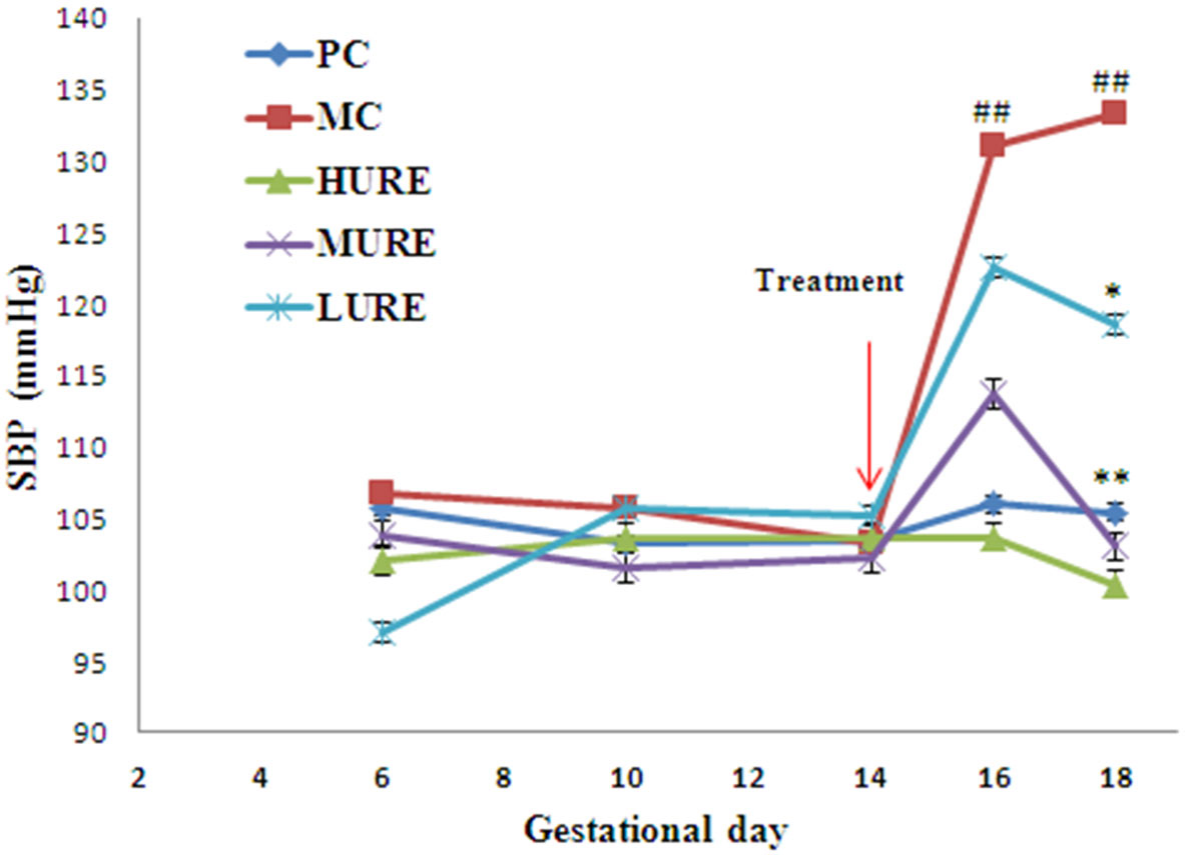
*Uncaria rhynchophylla* alkaloid extract (URE) reduced systolic blood pressures (SBP) in the lipopolysaccharide (LPS)-induced preeclampsia-like rats. SBP was measured on gestational days 6, 10, 14, 16, and 18 in the pregnant groups. Data are reported as means±SE. ^##^P<0.01 *vs* PC group, *P<0.05 and **P<0.01 *vs* MC group (ANOVA). PC: pregnant control group; MC: LPS-treated group; HURE: LPS plus high-dose URE; MURE: LPS plus medium-dose URE; LURE: LPS plus low-dose URE.

As displayed in Figure 3, SBP in the PC group did not change significantly during the study period. There were no differences in SBP profiles among the five groups before GD 14, but it was significantly increased in LPS-induced rats on GD 16 and 18 compared with the PC group (P<0.01). However, the values of SBP were significantly lower in the treated rats than those in the untreated rats (HURE *vs* MC, 100.33±5.85 mmHg *vs* 133.33±4.58 mmHg and MURE *vs* MC, 103.00±4.69 mmHg *vs* 133.33±4.58 mmHg on GD 18) (P<0.01).

### URE alleviated the proteinuria in LPS-induced PE-like rats

**Figure 4.**
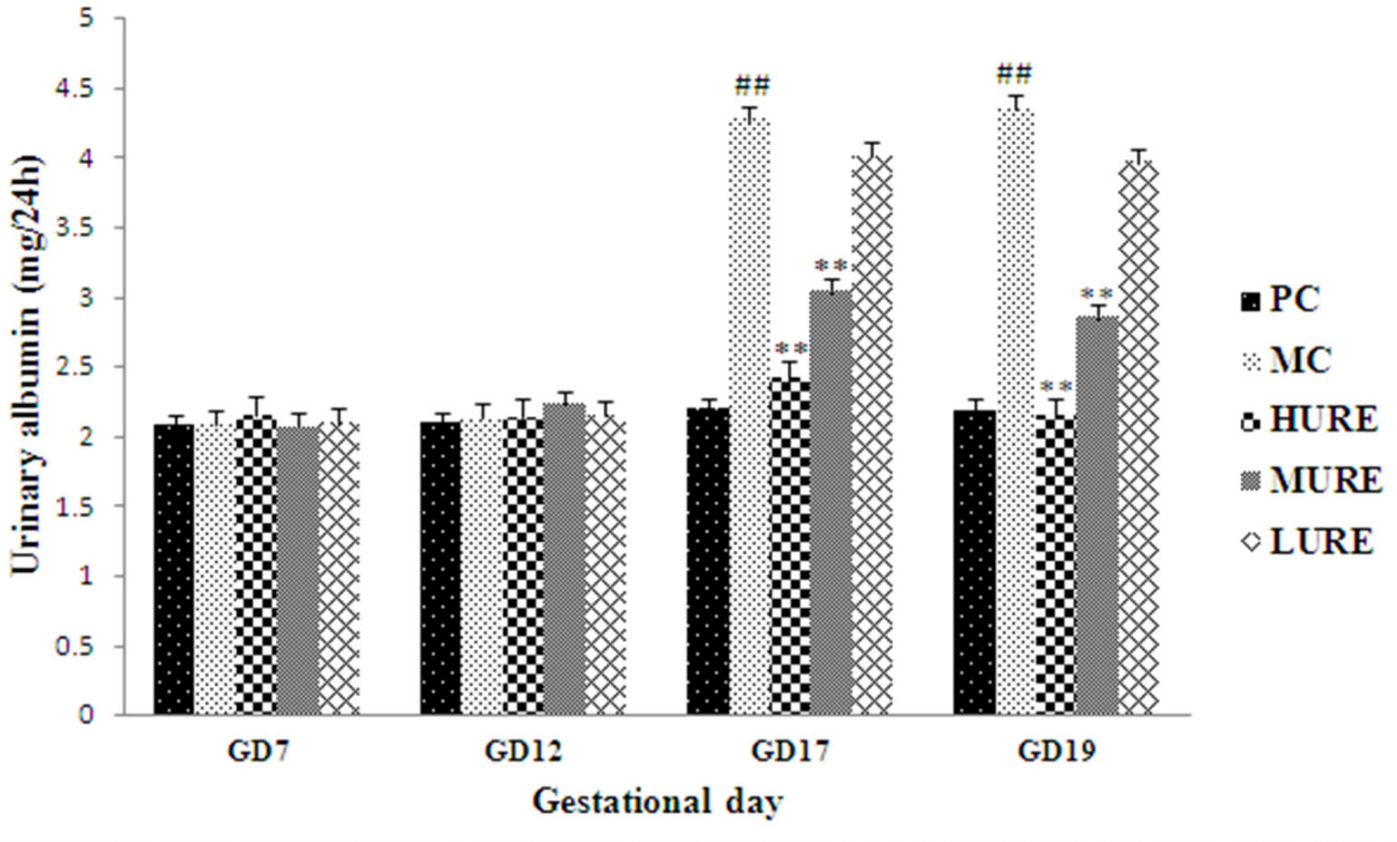
*Uncaria rhynchophylla* alkaloid extract (URE) ameliorated urinary albumin in the lipopolysaccharide (LPS)-induced preeclampsia-like rats. The 24-h urinary albumin was measured on gestational day (GD) 7, 12, 17, and 19. Data are reported as means±SE. ^##^P<0.01 *vs* PC group, **P<0.01 *vs* MC group (ANOVA). PC: pregnant control group; MC: LPS-treated group; HURE: LPS plus high-dose URE; MURE: LPS plus medium-dose URE; LURE: LPS plus low-dose URE.

As shown in Figure 4, there was no difference in proteinuria among the five groups before GD 14. The levels of 24-h proteinuria in the PC group did not change during the research period. However, levels of 24-h proteinuria were increased in the LPS-induced PE-like rats on GD 17 and 19 compared to those in the PC group (P<0.01). Interestingly, these differences were ameliorated by treatment with HURE and MURE (P<0.01).

### URE improved pregnancy outcomes in LPS-induced PE-like rats

As shown in Table 2, the placental weight, fetal weight, and number of live pups were significantly decreased in the MC group (P<0.05). However, HURE treatment increased placental weight, fetal weight, and number of live pups. These results indicated that URE can improve unfavorable pregnancy outcomes in LPS-induced PE-like rats.

**Table 2.** Pregnancy outcomes in different groups on gestational day 20.

Group	Number of live fetuses	Mean fetal weight (g)	Mean placental weight (g)
PC	9.7±0.8	4.9±0.24	0.54±0.02
MC	4.6±0.9^#^	3.7±0.35^#^	0.43±0.02^#^
HURE	10±1.3*	4.4±0.33	0.52±0.03
MURE	6.2±1.0	4.0±0.32	0.47±0.02
LURE	5.1±0.8	4.0±0.28	0.45±0.03

Data are reported as means±SE (n=8/group). ^#^P<0.05 *vs* the PC group, *P<0.05 *vs* the MC group (ANOVA). PC: pregnant control group; MC: lipopolysaccharide (LPS)-treated group; HURE: LPS plus high-dose *Uncaria rhynchophylla* alkaloid extract (URE); MURE: LPS plus medium-dose URE; LURE: LPS plus low-dose URE.

### Effects of URE on the levels of pro-inflammatory cytokines in the serum

**Figure 5.**
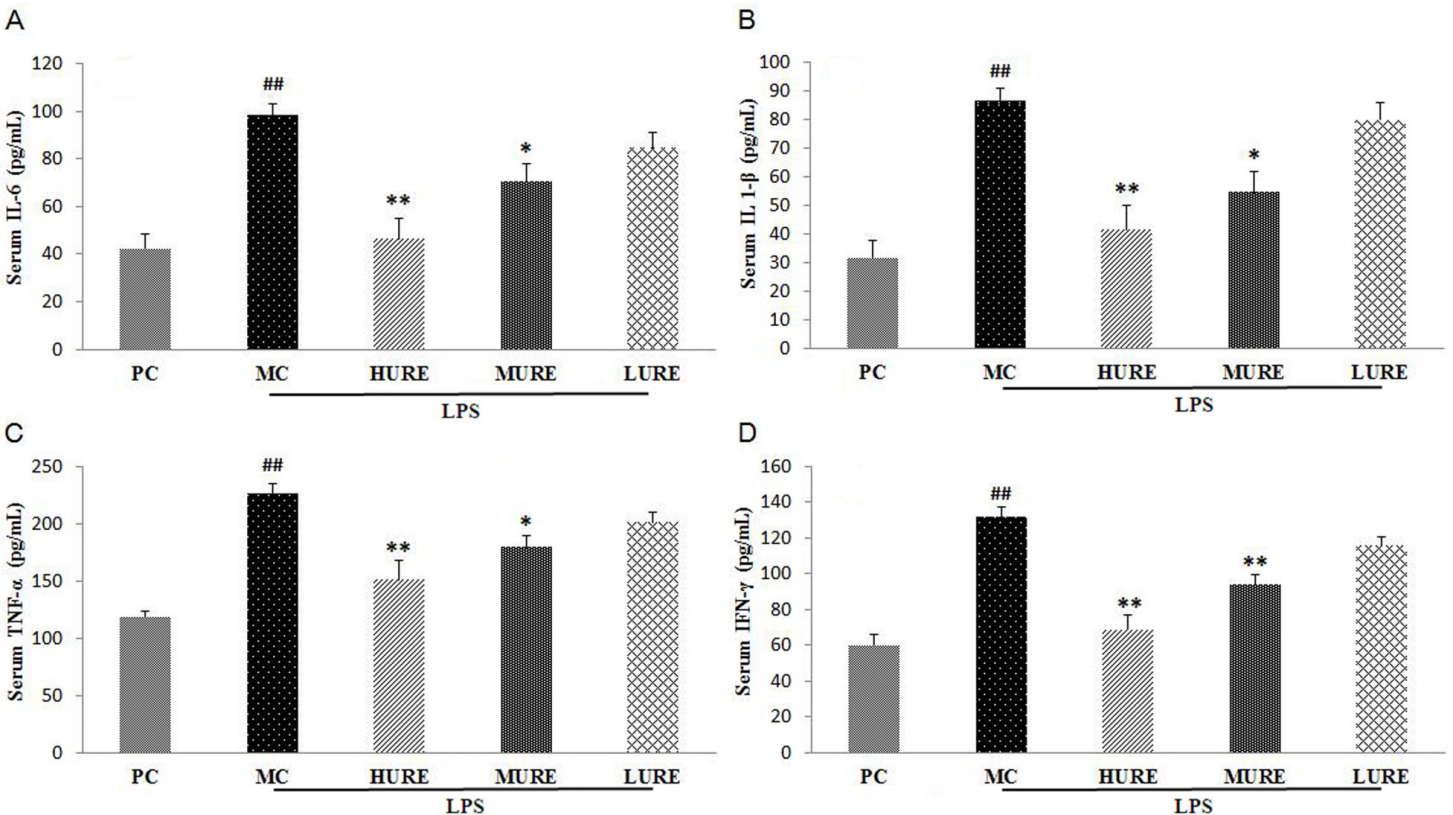
Serum levels of inflammatory cytokines in different groups on gestational day 20. Serum levels of interleukin (IL)-6 (**A**), IL 1-β (**B**), tumor necrosis factor (TNF)-α (**C**), and interferon (IFN)-γ (**D**) were significantly higher in the MC group and lower in the *Uncaria rhynchophylla* alkaloid extract (URE) group. Data are reported as means±SE (n=8/group). ^##^P<0.01 *vs* PC group, *P<0.05 and **P<0.01 *vs* MC group (ANOVA). PC: pregnant control group; MC: LPS-treated group; HURE: LPS plus high-dose URE; MURE: LPS plus medium-dose URE; LURE: LPS plus low-dose URE.

Data of serum pro-inflammatory cytokines are reported in Figure 5. After infusion of LPS, the levels of pro-inflammatory cytokines (IL-6, 98.45±8.45 pg/mL; IL 1-β, 86.65±5.93 pg/mL; TNF-α, 266.93±16.46 pg/mL; IFN-γ, 131.64±8.45 pg/mL) significantly increased in the MC group compared with the control group (P<0.01). The intragroup comparison showed that IL-6, IL-1β, TNF-α, and IFN-γ significantly declined after treatment with HURE compared with the MC group (P<0.01).

### Effects of URE on the levels of pro-inflammatory cytokines in the placenta

**Figure 6.**
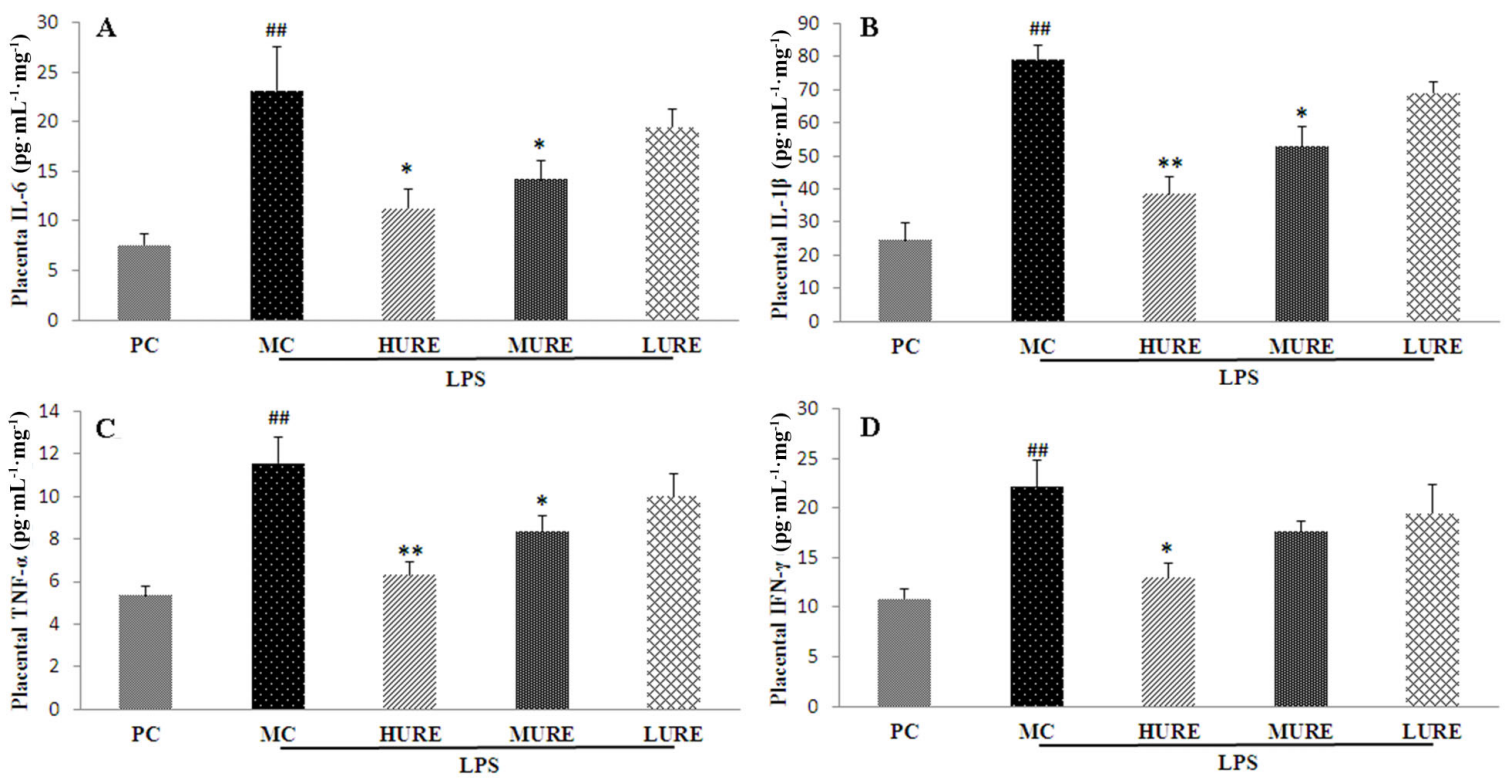
Placental levels of inflammatory cytokines in different groups on gestational day 20. Placental levels of interleukin IL-6 (**A**), IL1-β (**B**), tumor necrosis factor (TNF)-α (**C**), and interferon (IFN)-γ (**D**) were significantly higher in the MC group and lower in the *Uncaria rhynchophylla* alkaloid extract (URE) group. Data are reported as means±SE (n=8/group). ^##^P<0.01 *vs* PC group, *P<0.05 and **P<0.01 *vs* MC group (ANOVA). PC: pregnant control group; MC: LPS-treated group; HURE: LPS plus high-dose URE; MURE: LPS plus medium-dose URE; LURE: LPS plus low-dose URE.

Data of placenta pro-inflammatory cytokines are reported in Figure 6. After injection of LPS, the levels of pro-inflammatory cytokines (IL-6, 23.15±4.37 pg·mL^-1^·mg^-1^; IL 1-β, 79.15±4.32 pg· mL^-1^·mg^-1^; TNF-α, 11.57±1.19 pg· mL^-1^·mg^-1^; IFN-γ, 22.09±2.78 pg· mL^-1^·mg^-1^) in the MC group significantly increased compared with the control group (P<0.01). The intragroup comparison showed that IL-6, IL-1β, TNF-α, and IFN-γ significantly declined after treatment with HURE compared with the MC group (P<0.01).

### Pro-inflammatory cytokines mRNA expression

**Figure 7.**
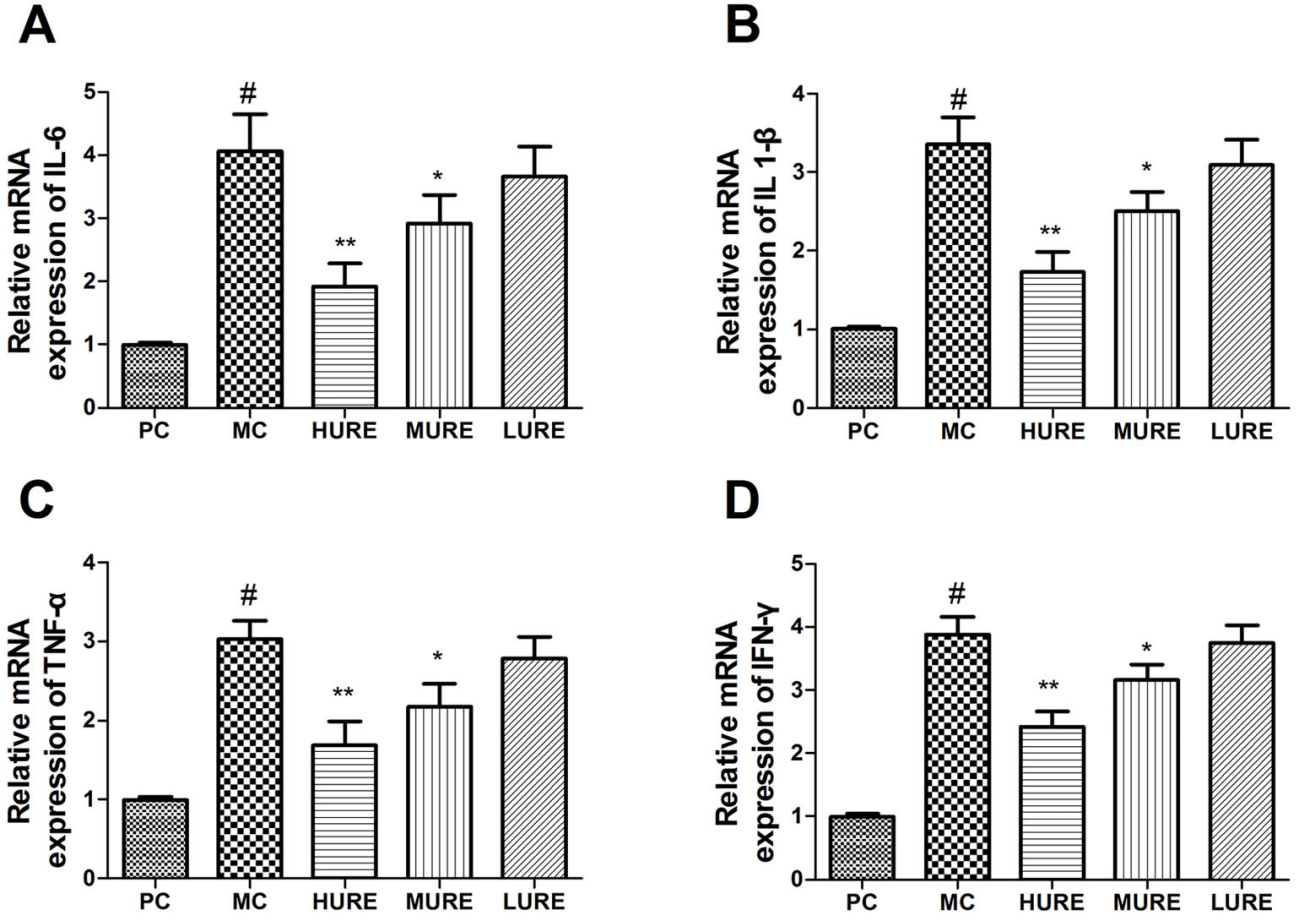
Relative mRNA levels of interleukin (IL)-6 (**A**), IL-1β (**B**), tumor necrosis factor (TNF)-α (**C**), and interferon (IFN)-γ (**D**) in placenta tissue determined by qPCR. Data are reported as means±SE (n=8/group). ^#^P<0.01 *vs* the PC group, *P<0.05 and **P<0.01 *vs* the MC group (ANOVA). PC: pregnant control group; MC: LPS-treated group; HURE: LPS plus high-dose *Uncaria rhynchophylla* alkaloid extract (URE); MURE: LPS plus medium-dose URE; LURE: LPS plus low-dose URE.

As shown in Figure 7, the mRNA expressions of IL-6, IL 1-β, TNF-α, and IFN-γ were up-regulated in the MC group compared to the NC group. However, HURE treatment down-regulated those expressions compared to the MC group (P<0.01). Accordingly, these data indicated that pro-inflammatory cytokines mRNA expressions were suppressed by HURE and thereby inflammatory response was mitigated in the LPS-treated rats.

### URE suppressed the LPS-induced activation of NF-**κ**B p65 in the placenta

**Figure 8.**
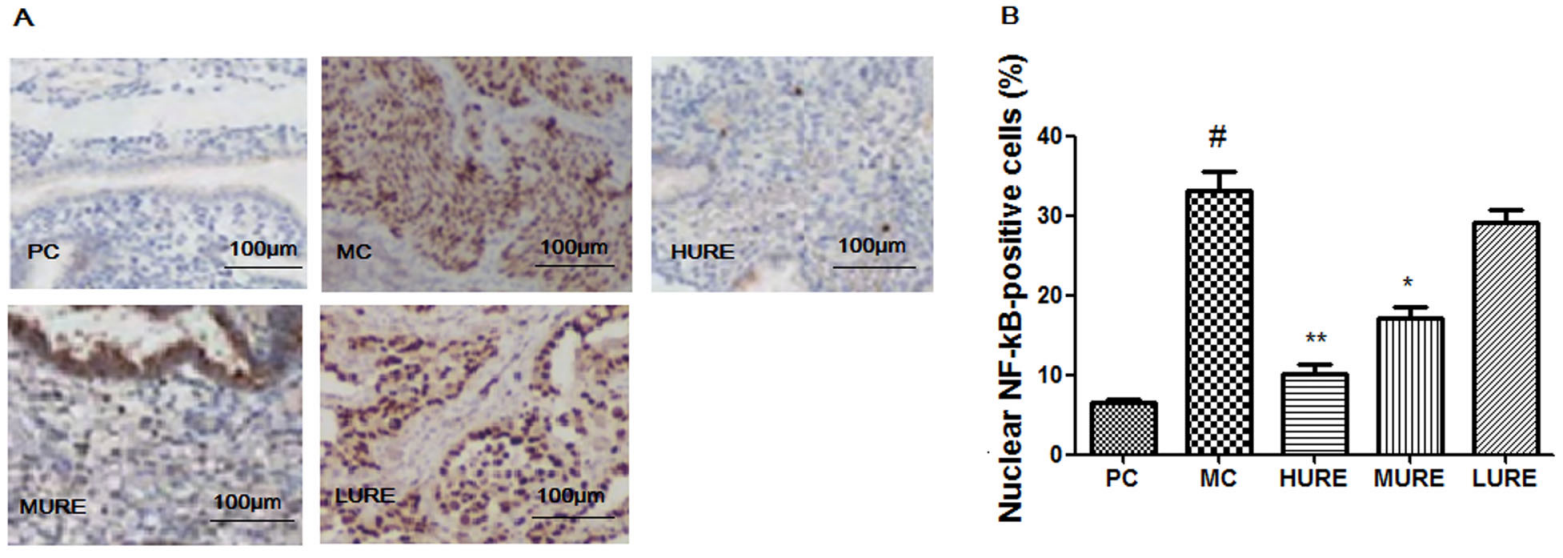
*Uncaria rhynchophylla* alkaloid extract (URE) suppressed the lipopolysaccharide (LPS)-induced activation of NF-κB p65 in the placenta tissue. Immunohistochemical staining of placental tissues (**A**). Original magnification: ×100; scale bar=100 μm. **B**, Percentage of nuclear NF-kB-positive cells in placenta tissue. Data are reported as means±SE (n=8/group). ^#^P<0.01 *vs* PC group, *P<0.05 and **P<0.01 *vs* MC group (ANOVA). PC: pregnant control group; MC: LPS-treated group; HURE: LPS plus high-dose URE; MURE: LPS plus medium-dose URE; LURE: LPS plus low-dose URE.

As shown in Figure 8, the level of NF-κB p65 was increased in the LPS-induced PE rats, whereas HURE treatment decreased nuclear-positive staining compared to the MC group (P<0.01).

## Discussion

Indole alkaloids are the main bioactive constituents presented in *U. rhynchophylla*, and play a vital role in improving anti-inflammatory effects and reducing hypertensive effects (12,22). However, there is no study linking the improvement by alkaloids from *U. rhynchophylla* to the PE-like phenotypes and pro-inflammatory cytokines caused by LPS. In this study, the purified URE improved adverse pregnancy outcomes by ameliorating PE-like symptoms and suppressing inflammation in the LPS-treated rats.

PE is characterized by increased maternal pro-inflammatory cytokines, including IL-6, IL-1β, TNF-α, and IFN-γ (7). Previous research has exhibited that excessive inflammation plays a critical role in the pathology and pathogenesis of PE (28). There are several pregnant rat models by injection of LPS, which is a potent virulence factor in terms of pro-inflammatory property (8,22). In the present study, a rat model of PE was successfully established by injecting LPS daily to pregnant rats until GD 14. The cause for the obvious elevation of blood pressure in LPS-treated rats may be due to excessive maternal pro-inflammatory cytokines resulting in alteration of maternal hemostasis, endothelial dysfunction, and vasoconstriction (29). In addition, LPS also increased the level of proteinuria in pregnant rats. Furthermore, the finding of declined fetal and placental weight and increased embryonic resorption in LPS-treated rats is in accordance with previous results in humans exhibiting fetal growth restriction and fetal loss in Gram-negative bacterial infections (30). These results imply that inflammation is an important mechanism in the pathogenesis of LPS-induced PE-like symptoms.

Accumulating evidence has shown that modulation of inflammation may be an effective therapy for the prevention and treatment of pregnancy complications. As vitamin D3 and aspirin possess anti-inflammatory effects, low-dose vitamin D3 and aspirin in early gestation were used for reducing the risk of PE and adverse perinatal health outcomes (31). The dried stems of *U. rhynchophylla* have been used as an antihypertensive, antipyretic, and anticonvulsant in traditional Chinese medicine. Research has also shown that *U. rhynchophylla* relieves atopic dermatitis (32) and the indole alkaloids of *U. rhynchophylla* have anti-inflammatory mediators such as IL-1β, NO, and TNF-α. In LPS-activated N9 cells, the anti-inflammatory mechanisms of alkaloids were relieved by blocking the activation of NF-κB, ERK, and p38 MAPKs (33). Due to its anti-inflammatory effect, *U. rhynchophylla* may be useful in the prevention and treatment of inflammation-related PE-like phenotypes.

The present research showed that URE treatment significantly prevented the emergence of phenotypes of PE in LPS-administered rats from GD14 to GD 18. Moreover, URE also improved pregnancy outcomes in the MC group, with increased fetal weight, placenta weight, and the number of live fetuses. These results suggested that URE may be an effective therapy for prevention and treatment of preeclampsia-like phenotypes and adverse pregnancy outcomes. Furthermore, in our LPS-induced rat model of PE, we found that URE effectively ameliorated urinary protein excretion and SBP, and that these changes were accompanied by suppression of the levels of pro-inflammatory cytokines in the placenta and serum. The present research supports that URE can alleviate PE and suppress inflammation to improve adverse pregnancy outcomes in rats.

The phytochemical analysis of the URE revealed the presence of indole alkaloids, including isorhynchophylline, yohimbine, 3α-dihydrocadambine, raubasine, hirsuteine, and hirsutine. Previous research has disclosed that indole alkaloids extracts are responsible for anti-inflammatory and anti-hypertensive activities, with which our experimental results are in accordance. Mitraphylline and isomitraphylline from *Uncaria tomentosa* extracts inhibited the production of pro-inflammatory cytokines *in vitro* (34). Rhynchophylline and isorhynchophylline from *U. rhynchophylla* extracts suppressed inflammatory mediators by blocking iNOS protein expression and blocking the activation of NF-κB, ERK, and p38 MAPKs in N9 cells (33). 3α-dihydrocadambine showed dose-dependent anti-hypertensive and hypotensive activities in conscious spontaneous rats and in anesthetized normotensive rats (35).

In conclusion, our research showed that URE improved PE signs and inhibited pro-inflammatory cytokines in a PE rat model. URE is a native medicine with anti-inflammatory and anti-hypertensive properties, which could be attributed to indole alkaloids. These findings revealed a novel therapeutic use of URE for preventing excessive pro-inflammatory cytokines in pregnancy complications. However, the underlying mechanisms need to be elucidated in further research.
